# Supplementary material for: Surveillance of Antimicrobial Resistance in the ECOWAS Region: Setting the Scene for Critical Interventions Needed
Source: Antibiotics (Basel). 2024 Jul 5;13(7):627. doi: 10.3390/antibiotics13070627 (PMC11273779; doi:10.3390/antibiotics13070627)
Supplement: Supplementary file 1 [file antibiotics-13-00627-s001.zip › antibiotics-3078332-supplementary.pdf]

Table S1: National surveillance system for antimicrobial resistance (AMR) in humans, capacity in 2018 and 2022

**Country name**	**National surveillance system for antimicrobial resistance (AMR) in humans**	
	2018	2022
Côte d'Ivoire	B - AMR data is collated locally for common bacteria, but data collection may not use a standardized approach and lacks national coordination and/or quality management.	D - There is a standardized national AMR surveillance system collecting data on common bacterial infections in hospitalized and community patients, with established network of surveillance sites, designated national reference laboratory for AMR, and a national coordinating centre producing reports on AMR.
Ghana	B - AMR data is collated locally for common bacteria, but data collection may not use a standardized approach and lacks national coordination and/or quality management.	D - There is a standardized national AMR surveillance system collecting data on common bacterial infections in hospitalized and community patients, with established network of surveillance sites, designated national reference laboratory for AMR, and a national coordinating centre producing reports on AMR.
Benin	B - AMR data is collated locally for common bacteria, but data collection may not use a standardized approach and lacks national coordination and/or quality management.	C - AMR data are collated nationally for common bacterial infections in hospitalized and community patients, but national coordination and standardization are lacking.
Burkina Faso	C - National AMR surveillance activities for common bacterial infections follow national standards, and a national reference laboratory that participates in external quality assurance.	D - There is a standardized national AMR surveillance system collecting data on common bacterial infections in hospitalized and community patients, with established network of surveillance sites, designated national reference laboratory for AMR, and a national coordinating centre producing reports on AMR.
Guinea	B - AMR data is collated locally for common bacteria, but data collection may not use a standardized approach and lacks national coordination and/or quality management.	C - AMR data are collated nationally for common bacterial infections in hospitalized and community patients, but national coordination and standardization are lacking.
Sierra Leone	A - No capacity for generating data (antibiotic susceptibility testing and accompanying clinical and epidemiological data) and reporting on antibiotic resistance.	B - AMR data is collated locally for common* bacterial infections in hospitalized and community patients* , but data collection may not use a standardized approach and lacks national coordination and/or quality management.
Togo	B - AMR data is collated locally for common bacteria, but data collection may not use a standardized approach and lacks national coordination and/or quality management.	C - AMR data are collated nationally for common bacterial infections in hospitalized and community patients, but national coordination and standardization are lacking.
Liberia	B - AMR data is collated locally for common bacteria, but data collection may not use a standardized approach and lacks national coordination and/or quality management.	B - AMR data is collated locally for common* bacterial infections in hospitalized and community patients* , but data collection may not use a standardized approach and lacks national coordination and/or quality management.
Mali	D - There is a functioning national AMR surveillance system covering common bacterial infections in hospitalized and community patients+, with external quality assurance, and a national coordinating centre producing reports on AMR.	D - There is a standardized national AMR surveillance system collecting data on common bacterial infections in hospitalized and community patients, with established network of surveillance sites, designated national reference laboratory for AMR, and a national coordinating centre producing reports on AMR.
Nigeria	D - There is a functioning national AMR surveillance system covering common bacterial infections in hospitalized and community patients+, with external quality assurance, and a national coordinating centre producing reports on AMR.	D - There is a standardized national AMR surveillance system collecting data on common bacterial infections in hospitalized and community patients, with established network of surveillance sites, designated national reference laboratory for AMR, and a national coordinating centre producing reports on AMR.

Table S2: AMR reference laboratory capacities

	Does the country have one or more reference lab/s performing AST/susceptibility testing for all the bacteria listed	Acinetobacter baumannii	Pseudomonas aeruginosa	Enterobacteriaceae E.coli and Klebsiella, Proteus	Enterococcus faecium	Staphylococcus aureus	Campylobacter spp.	Salmonellae	Neisseria gonorrhoeae	Streptococcus pneumoniae	Haemophilus influenzae	Shigella spp.
Guinea	Yes, ALL the 11 bacteria listed	Yes	Yes	Yes	Yes	Yes	Yes	Yes	Yes	Yes	Yes	Yes
Mali	Yes, ALL the 11 bacteria listed	Yes	Yes	Yes	Yes	Yes	Yes	Yes	Yes	Yes	Yes	Yes
Guinea-Bissau	Yes, ALL the 11 bacteria listed	Yes	Yes	Yes	Yes	Yes	Yes	Yes	Yes	Yes	Yes	Yes
Ghana	Yes, some of the bacteria listed;	Yes	Yes	Yes	Yes	Yes	X	Yes	Yes	Yes	Yes	Yes
Sierra Leone	Yes, ALL the 11 bacteria listed	Yes	Yes	Yes	Yes	Yes	Yes	Yes	Yes	Yes	Yes	Yes
Nigeria	Yes, some of the bacteria listed;	Yes	Yes	Yes	Yes	Yes	Yes	Yes	No	Yes	Yes	Yes
Senegal	Yes, ALL the 11 bacteria listed	Yes	Yes	Yes	Yes	Yes	Yes	Yes	Yes	Yes	Yes	Yes
Benin	Yes, ALL the 11 bacteria listed	Yes	Yes	Yes	Yes	Yes	Yes	Yes	Yes	Yes	Yes	Yes
Liberia	Yes, ALL the 11 bacteria listed	Yes	Yes	Yes	Yes	Yes	Yes	Yes	Yes	Yes	Yes	Yes
Côte d'Ivoire	Yes, ALL the 11 bacteria listed	Yes	Yes	Yes	Yes	Yes	Yes	Yes	Yes	Yes	Yes	Yes
Burkina Faso	Yes, some of the bacteria listed;	Yes	Yes	Yes	Yes	Yes	X	Yes	Yes	Yes	Yes	Yes
Cabo Verde	No, the country does not have a reference lab/s performing susceptibility testing for any of the bacteria	X	X	X	X	X	X	X	X	X	X	X
Niger	No reponse	X	X	X	X	X	X	X	X	X	X	X
Togo	Yes, some of the bacteria listed;	Yes	Yes	Yes	Yes	Yes	X	Yes	Yes	Yes	Yes	Yes

Table S3: AMR NAPs integration

	Is your country's national planning on AMR integrated with other existing action plans or, strategies?	National health sector plan	National action plan on health security	One Health Strategy or One Health mechanism	Water, Sanitation and Hygiene (WASH)	Climate change and environmental planning	National Agriculture development plans and policies	United Nations Sustainable Development Coorporation Framework (UNSDCF)	National Food Safety strategy and policies
Guinea	Yes	Yes	Yes	Yes	Yes	Yes	Yes	No	No
Mali	Yes	No	Yes	No	No	No	No	No	No
Guinea-Bissau	Yes	Yes	No	Yes	Yes	No	No	No	No
Ghana	Yes	No	Yes	No	Yes	Yes	No	No	No
Sierra Leone	Yes	Yes	Yes	Yes	Yes	Yes	Yes	Yes	Yes
Nigeria	Yes	No	Yes	Yes	Yes	No	No	No	Yes
Senegal	Yes	Yes	Yes	Yes	Yes	Yes	Yes	Yes	Yes
Benin	Yes	No	No	Yes	No	No	No	No	No
Liberia	Yes	Yes	Yes	Yes	Yes	No	No	Yes	No
Côte d'Ivoire	Yes	Yes	Yes	Yes	Yes	No	No	No	No
Burkina Faso	Yes	Yes	No	Yes	No	No	No	No	No
Cabo Verde	Yes	Yes	Yes	No	No	No	Yes	No	No
Niger	Yes	Yes	No	No	Yes	No	No	No	No
Togo	Yes	Yes	Yes	No	No	No	No	No	No
Summary									
Yes	14	10	10	9	9	4	4	3	3
No	0	4	4	5	5	10	10	11	11
